# High-Precision MEMS Resonant Pressure Sensor for Real-Time Barometric Monitoring

**DOI:** 10.3390/mi17060717

**Published:** 2026-06-12

**Authors:** Fei Xia, Shuang Pang, Yutong Bai, Zishuai Zhang, Lulu Feng, Yizheng Hou, Yuxiang Wang, Zhiyu Liu, Yifei Sun, Jiwei Wang, Shiyu Wang

**Affiliations:** 1 College of Physics, Liaoning University, Shenyang 110036, China; 13555888537@163.com (F.X.); pangshuangemail@163.com (S.P.); 18333505225@163.com (Y.B.); z1061897905@163.com (Z.Z.); ll110810278@163.com (L.F.); 15904289700@163.com (Y.H.); meteoro1029wyx@163.com (Y.W.); l2579361117@163.com (Z.L.); wangjiwei@lnu.edu.cn (J.W.); 2 College of Information Engineering, Shenyang University of Chemical Technology, Shenyang 110142, China; sunyifei@syuct.edu.cn

**Keywords:** MEMS pressure sensor, resonant sensor, electrostatic excitation, piezoresistive detection, real-time barometric monitoring

## Abstract

Addressing the urgent demand for high-precision pressure measurement in real-time barometric monitoring, aerospace, and industrial control, this paper presents a high-accuracy MEMS resonant pressure sensor based on electrostatic excitation and piezoresistive detection. The sensor incorporates a symmetric double-ended fixed-finger comb-drive resonator structure, driven into stable vibration at its natural frequency by an alternating electrostatic force. Piezoresistors integrated at the root of the resonant beams transduce the mechanical vibration into a frequency output, enabling precise external pressure measurement. Experimental results show that the developed sensor achieves an accuracy of 0.009% FS over a pressure range of 0–350 kPa across an operating temperature span from −30 °C to 50 °C, with a room-temperature repeatability error below 0.008% FS, demonstrating excellent measurement stability. Building on this performance, a real-time atmospheric pressure monitoring experiment was conducted, yielding a mean absolute percentage error of less than 0.05%, highlighting the sensor’s potential for engineering practicality. This work provides an effective technique for a high-precision, high-stability resonant pressure sensor, with clear potential for deployment in real-time barometric monitoring, aerospace, and industrial control applications.

## 1. Introduction

With the ongoing refinement and intelligent upgrading of industrial manufacturing, along with the continuous expansion of extreme application scenarios such as high-altitude sounding and high-temperature oil and gas extraction, the global demand for pressure measurement instruments that combine ultra-high precision with long-term stability has become increasingly urgent [1,2,3].

Among the various types of pressure sensors, MEMS resonant pressure sensors are recognized as a preferred solution for precision pressure measurement because their output is in the form of a digital frequency signal, which offers strong immunity to electromagnetic interference and facilitates seamless interfacing with digital systems [4,5,6,7,8]. Recent studies have further advanced the performance of MEMS resonant pressure sensors. Xia et al. developed a single-resonator sensor with amplitude-based temperature compensation, achieving ±0.012% FS accuracy over −20 °C to 60 °C [9]; Lu et al. demonstrated an oil-filled sensor with electrostatic stiffness modulation, reaching ±0.02% FS from −55 °C to 125 °C [10]; Li et al. reported a high-Q resonant differential pressure sensor using wafer-level through-glass-via packaging, achieving an accuracy better than 0.027% FS over −45 °C to 45 °C [11]. These studies demonstrate good potential for practical applications. However, existing MEMS resonant pressure sensors still face several common technical challenges in engineering applications. On the one hand, electromagnetic excitation and detection schemes involve complex structures that are difficult to integrate [12]. On the other hand, the combination of electrostatic excitation and capacitive detection is highly sensitive to parasitic capacitances, imposing stringent requirements on the design of signal conditioning circuits [13,14]. Consequently, achieving a balance between structural simplicity of the sensor and reliability of signal detection remains a focal point of research in this field [15].

The sensor development is achieved through a co-design strategy that jointly optimizes the mechanical resonator structure and the dedicated interface circuitry, realizing a MEMS resonant pressure sensor based on electrostatic excitation and piezoresistive detection. In terms of resonator structure design, a symmetric double-ended fixed-finger comb-drive resonator configuration is employed. During vibration, the two beams oscillate in opposite directions within the plane, forming a dynamically balanced mode that effectively reduces energy dissipation and minimizes the influence of overall device vibration on measurement results [16,17]. Regarding hardware circuitry and loop compensation, a feedforward compensation technique is implemented to effectively eliminate startup waveform overshoot, thereby significantly shortening the system stabilization time.

Benefiting from the innovative designs described above, the fabricated electrostatic excitation and piezoresistive pickup MEMS resonant pressure sensor achieves an accuracy of 0.009% FS over the pressure range of 0–350 kPa across an operating temperature window from −30 °C to 50 °C. The sensor also exhibits a repeatability error of less than 0.008% FS, demonstrating excellent measurement precision and temperature robustness. Furthermore, a week-long real-time atmospheric pressure monitoring experiment was carried out. The results show a mean absolute percentage error of less than 0.05% compared with reference atmospheric pressure data for Shenyang, China, provided by the China Meteorological Administration. This outcome confirms the high accuracy and reliability of the pressure sensing system in practical atmospheric pressure measurement. The present study provides a high-precision, high-stability MEMS resonant pressure sensing solution, which features a symmetric dynamically balanced resonator, a feedforward compensation circuit, and a third-order polynomial temperature compensation algorithm. It has broad application potential in critical fields such as industrial automation, aerospace, energy exploration, and barometric monitoring.

## 2. Materials and Methods

### 2.1. Overall Architecture

**Figure 1:**
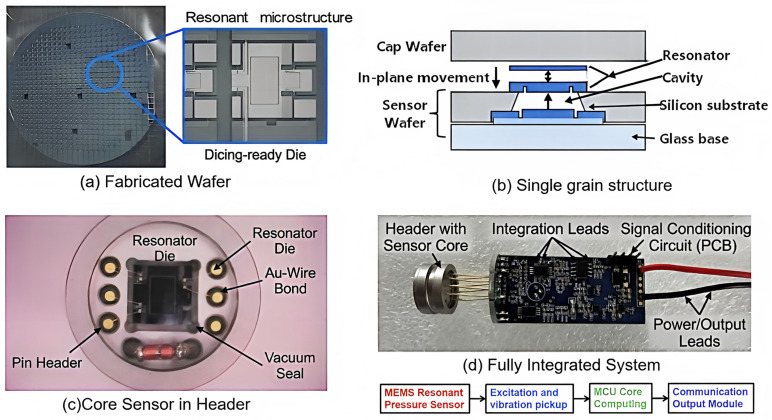
Schematic diagram of the overall architecture of the MEMS resonant pressure sensing system. (**a**) Photograph of the fabricated wafer. (**b**) Three-dimensional structure of a single MEMS chip. (**c**) Photograph of the core MEMS sensor assembled on a metal header. (**d**) Fully integrated system module of the MEMS resonant pressure sensor.

A high-precision resonant pressure sensor was designed in this work, adopting a modular system architecture primarily composed of three functional blocks: a MEMS resonant pressure sensing module, an excitation/pickup and MCU core processing module, and a communication output module. The system block diagram is illustrated in Figure 1. Specifically, Figure 1a shows the MEMS resonant pressure sensor wafer, Figure 1b presents the three-dimensional structure of an individual MEMS chip, Figure 1c displays the package assembly of the MEMS resonant pressure sensing module, and Figure 1d depicts the overall system structure and module schematic.

Figure S1 further illustrates the detailed signal processing and data conversion flow of the system. First, the DC12V power supply is filtered and converted to generate DC10V (for the excitation circuit), DC5V (for operational amplifiers), and DC3.3V (for the MCU). Subsequently, the pickup circuit performs phase shifting and amplification of the piezoresistive signal, and a multi-stage feedback and voltage-controlled gain loop achieves stable amplitude oscillation. The MCU core module then applies algorithmic compensation and pressure calculation to the frequency signal, and finally outputs a standard digital signal via the RS485 interface. This flow outlines the complete signal chain from physical sensing to digital output, which serves as a reference for the module-level discussions that follow.

The excitation and pickup circuitry, the MCU core processing module, and the communication output module are highly integrated onto a single embedded printed circuit board (PCB) to minimize system volume and enhance signal integrity. The corresponding PCB layout and circuit schematic diagrams are provided in Figures S2 and S3, respectively.

During system operation, the excitation circuit drives the resonator within the MEMS resonant pressure sensing module to generate stable mechanical vibration. The pickup circuit detects this vibration frequency in real time and converts it into an electrical signal. When external pressure is applied to the sensing module, the natural frequency of the resonator shifts accordingly. The MCU core processing module acquires the frequency signal, performs computation, and inversely determines the applied pressure value. Finally, the calculated result is transmitted via the communication output module through an RS485 interface (Modbus protocol) to an interactive output terminal, enabling user interaction functions such as data display, parameter configuration, and system monitoring. The detailed design and operational principles of each aforementioned module are elaborated in Section 2.2, Section 2.3 and Section 2.4.

### 2.2. Design of the MEMS Resonant Pressure Sensing Module

**Figure 2:**
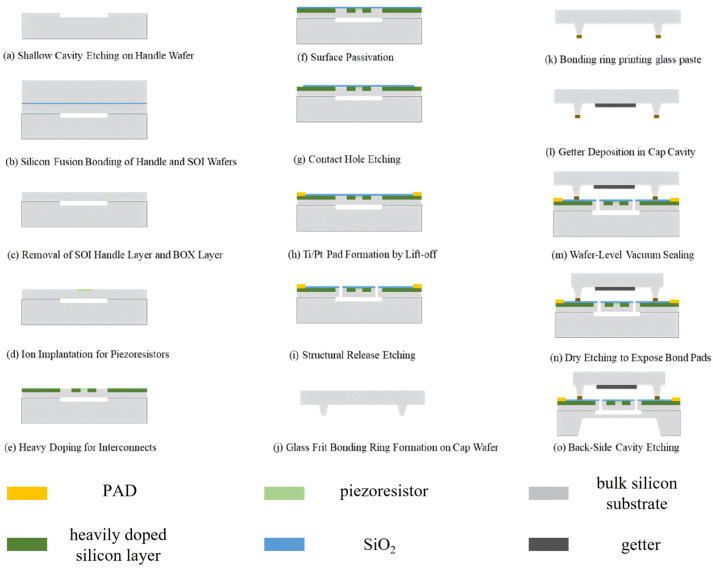
Detailed fabrication process flow of the MEMS resonant pressure sensing module.

A MEMS resonant pressure sensing module based on silicon-on-insulator (SOI) wafer technology was designed and fabricated in this work [18,19,20]. The complete list of key specifications, including mechanical, electrical, and environmental parameters, is provided in Table S1 of the Supporting Information. The core structure consists of three components arranged from top to bottom: an SOI sensing chip, a glass substrate with a pre-etched cavity, and a metal header. The detailed fabrication process flow is illustrated in Figure 2.

**Figure 3:**
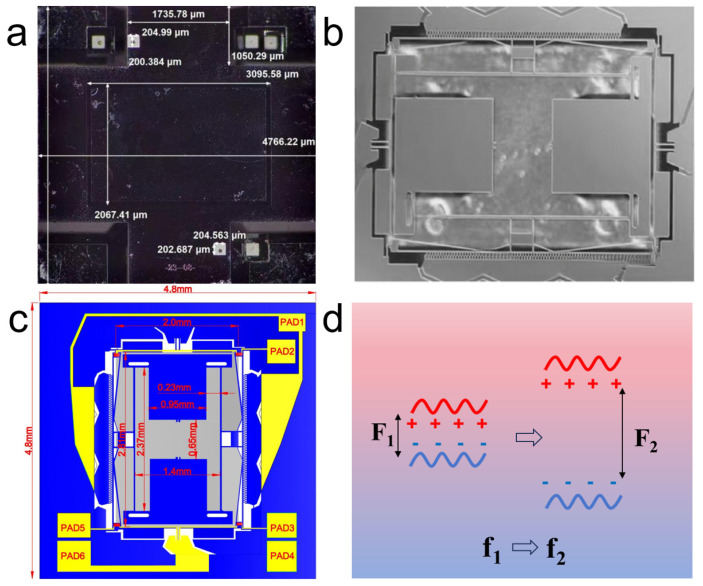
(**a**) Overall structure of the MEMS resonant pressure sensing module. (**b**,**c**) Cross-sectional structural diagrams of the SOI sensing chip. In Figure 3c, the piezoresistive regions are shown in red, and the electrical routing paths are in yellow. The electrostatic drive terminal is labeled PAD1, the piezoresistive pick-up terminal is PAD6, and PAD2/PAD3/PAD4/PAD5 are common ground terminals. Only two symmetrically located piezoresistors (near PAD3 and PAD5) are used; they are connected in parallel for piezoresistive detection. (**d**) Detection principle schematic of the MEMS resonant pressure sensing module.

The SOI sensing chip integrates the critical pressure-sensitive diaphragm and resonator structures. The glass substrate is bonded to the SOI sensing chip via anodic bonding, collectively forming a stable vacuum reference cavity. Figure 3a depicts the overall structure of the MEMS resonant pressure sensing module after anodic bonding. The resonators are symmetrically distributed on the upper and lower regions of the chip, with comb-drive structures employed to apply electrostatic forces to the resonant beams. The excitation signal combines a DC bias voltage (Vdc) and an AC sinusoidal voltage (Vac). The DC bias establishes the electrostatic stiffness and a constant electrostatic force, while the AC component provides the alternating force that sustains mechanical resonance. The comb-drive electrodes are electrically connected through the heavily doped silicon layer (no metal deposition on the comb fingers). The structural diagrams of the SOI sensing chip are presented in Figure 3b,c. When external pressure acts upon the pressure-sensitive diaphragm, the diaphragm undergoes bending deformation, which displaces the coupled comb-drive structures. The electrostatic stiffness is defined as the negative derivative of the electrostatic force with respect to displacement, which depends sensitively on the gap spacing. This displacement modulates the electrostatic stiffness experienced by the resonator, consequently shifting its natural frequency. By detecting this frequency change, the applied external pressure can be inversely determined. The corresponding detection principle is schematically illustrated in Figure 3d. This structural design effectively ensures a stable sensor response and long-term reliability across a wide pressure range [21,22,23].

### 2.3. Design of the Excitation and Pickup Circuit Module

The MEMS resonant pressure sensing module requires electrostatic excitation to initiate oscillation and relies on closed-loop automatic gain control (AGC) to stabilize the vibration at its natural frequency. Moreover, the piezoresistive output signal is inherently weak and susceptible to power supply noise and electromagnetic interference. Consequently, dedicated excitation and pickup circuitry must be designed. The excitation and pickup circuit presented in this work comprises two core functional blocks: the pickup circuit module and the excitation circuit module.

The pickup circuit module primarily consists of a pickup power supply circuit and a pickup/phase-shifting amplification circuit (Figure S4). In the pickup power supply circuit, a precision voltage reference provides a constant DC bias voltage (DC 5 V) for the silicon piezoresistors integrated within the MEMS resonant pressure sensing module, thereby minimizing the impact of supply fluctuations on sensitivity and stability. Within the pickup and phase-shifting amplification circuit, resistor R38 is employed to set the DC bias level and is matched to the resistance of the silicon piezoresistive element to maximize the extracted pickup signal. Precision operational amplifiers (U1, U2) are configured to amplify the weak detected signal, with adjustable gain settings to achieve an optimal level. Phase compensation of the resonant signal is accomplished through lead compensation capacitors (C38, C42) and DC blocking capacitors (C37, C40). The amplified resonant signal is subsequently output via port S1 to terminal P1A2 of the excitation circuit module.

The excitation circuit module mainly consists of an excitation power supply circuit and a multi-stage feedback/voltage-controlled gain amplification circuit (Figure S5). In the excitation power supply circuit, a precision voltage reference supplies a stable DC bias voltage and multiple operational amplifier reference voltages (DC bias voltage T2 = 10.0 V, reference voltage VREF = 2.5 V) to mitigate the influence of supply variations on excitation stability. Within the multi-stage feedback and voltage-controlled gain amplification circuit, a variable gain amplifier (VGA) architecture based on a JFET voltage-controlled resistor is implemented to achieve closed-loop automatic gain control of the excitation signal amplitude. The final DC bias voltage and excitation signal are fed to the excitation terminal S2 (P1).

### 2.4. Design of the MCU Core Processing and Output Module

Following system initialization, the MCU core processing module assumes responsibility for signal processing and pressure computation. Specifically, the module acquires the front-end conditioned frequency digital signal, performs filtering and noise reduction, and subsequently calculates the pressure value based on a pre-calibrated inverse transfer function. The computed result is then transmitted via RS485 (Modbus protocol) to the interactive output module.

The interactive output module converts the processed pressure data into industry-standard output signals and enables parameter management and system monitoring through a user interface. This module supports both RS485 digital communication and 4–20 mA analog current output, ensuring reliable interfacing with industrial control systems. Additionally, through a touchscreen interface and the Modbus protocol, the module facilitates the configuration of sensor calibration parameters and the real-time display of system operational status.

### 2.5. Performance Testing

**Figure 4:**
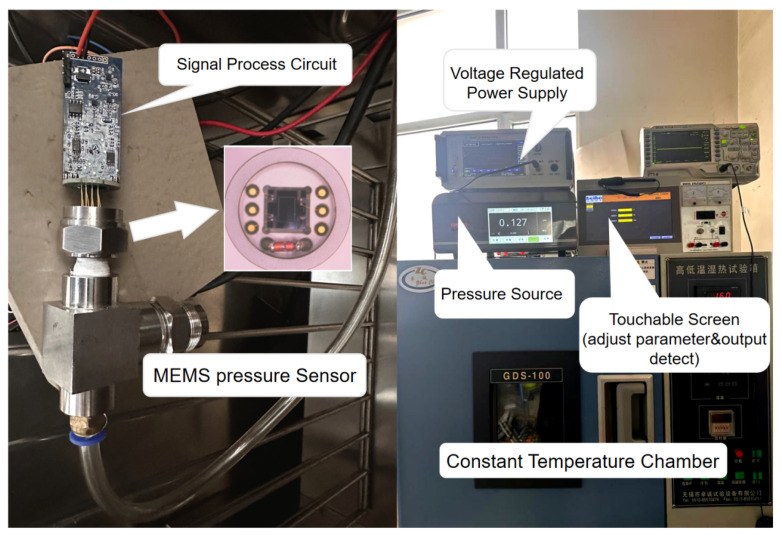
Temperature–frequency combined test platform for the MEMS resonant pressure sensing system.

To systematically evaluate the measurement accuracy, stability, and reliability of the MEMS resonant pressure sensor over a wide temperature range, a specialized electrical test platform was established, as illustrated in Figure 4. The core of this platform comprises high-precision pressure excitation, precise environmental simulation, multi-parameter data acquisition, and automated monitoring subsystems. Specifically, a WIKA CPC6050 standard pressure controller) provides accurate pressure excitation, while a GPS-3 high-low temperature chamber (temperature range: −70 °C to 150 °C, stability: ±0.5 °C) is employed to simulate operating environments from −30 °C to 50 °C. The sensor output signal is acquired using a Keysight DMM6500 6½-digit digital multimeter (Keysight Technologies Co., Ltd., Shanghai, China.), and power is supplied by a Keithley 2280S-60-3 precision DC power supply (Keysight Technologies Co., Ltd., Shanghai, China.). Real-time data monitoring and interaction are achieved via Modbus debugging software (McgsPro 3.5.1.6963 and Keil μVision V5.43.1.0) and a TPC7031NI touchscreen interface. During all actual tests, the sensor was first placed inside the temperature chamber and allowed to stabilize for 30 min at the target temperature point. Subsequently, standard pressure was applied using the pressure controller, and the sensor frequency output was synchronously recorded for performance evaluation. This testing protocol ensures controlled experimental conditions and reliable data acquisition, thereby establishing a solid foundation for subsequent performance analysis [24].

### 2.6. Atmospheric Pressure Measurement Application

To validate the numerical stability and accuracy of the developed pressure sensing system under actual field conditions, an atmospheric pressure measurement application experiment was conducted. During the experiment, the sensing system was placed in a well-ventilated location and directly exposed to the ambient atmospheric pressure. The consistency between the system’s measured values and the standard reference values was evaluated by comparing the acquired data with real-time local meteorological station data published by the China National Meteorological Science Data Center (https://data.cma.cn/).

The experiment was carried out on the campus of Liaoning University (Chongshan Campus) in Shenyang, China, from 10 April to 16 April 2026. The sensing system output the measured atmospheric pressure values to a computer in real time via a serial interface at a sampling rate of 1 Hz. The local atmospheric pressure values provided by the meteorological station were updated every five minutes. Accordingly, at each update time point, a total of 60 consecutive data points were acquired within a 30 s window both before and after the update moment. After removing obvious outliers, the arithmetic mean was calculated to obtain the raw measured atmospheric pressure value at that specific time instant.

## 3. Results

### 3.1. Wafer-Level Electrical Characterization of the MEMS Resonant Pressure Sensing Module

**Figure 5:**
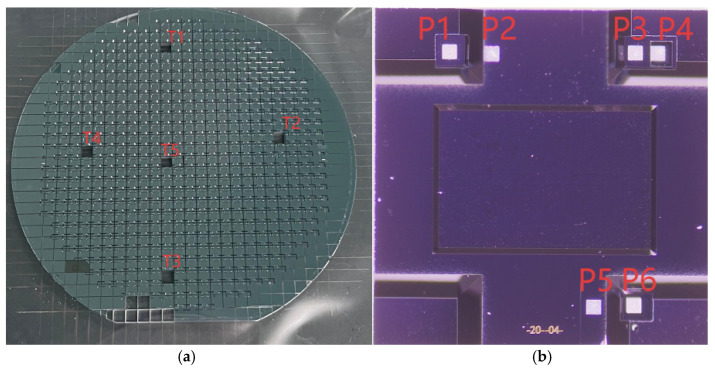
Wafer sampling and test point locations for the MEMS resonant pressure sensing wafer. (**a**) Layout of the sampled chip positions across the wafer. (**b**) Enlarged view of the test pad locations on a single chip.

Wafer-level electrical characterization is a critical step in ensuring device performance consistency and reliability [25]. For resonant pressure sensors, whose operating principle relies on the mechanical vibration modes of the resonant structure, the resistance uniformity of the piezoresistive elements within the sensitive structure and the electrical isolation state of the drive structures directly determine whether the sensor can be properly excited and output a stable frequency signal. To evaluate the process uniformity and electrical performance of the MEMS resonant pressure sensor wafer, a stratified random sampling method was adopted in this study. Five chips were selected as test samples from the center, edge, and intermediate regions of the wafer, labeled T1 through T5, respectively. The test results are presented in Figure 5. Specifically, the wafer-level electrical testing comprised the following two parts.

(1) Piezoresistance Measurement (Test Path: P4–P6)

**Figure 6:**
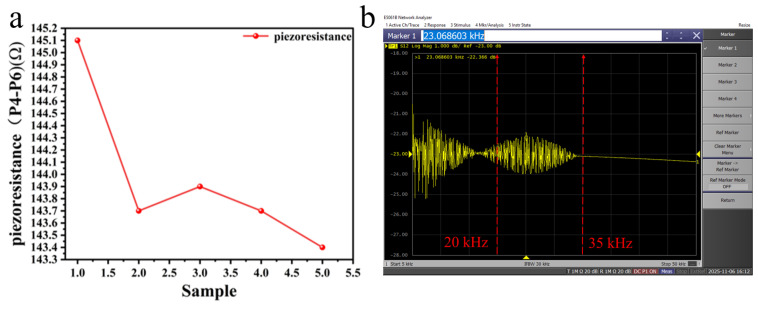
Test results: (**a**) Piezoresistance measurement results of the MEMS resonant pressure sensing wafer; (**b**) Frequency sweep test result of the MEMS resonant pressure sensor over the range of 5–50 kHz.

The objective of the piezoresistance measurement is to evaluate the uniformity of the piezoresistor doping process on the resonant beams. In this study, the four-probe method was employed. Under ambient conditions of room temperature (25 °C) and 45% relative humidity, a high-precision source meter Keithley 2400 (Keysight Technologies Co., Ltd., Shanghai, China.) was used to apply a constant current across the two-terminal electrodes of the piezoresistive element (test path P4–P6). The voltage drop was measured to calculate the resistance value, and each measurement was repeated three times with the average value taken to minimize systematic errors. The measurement results are shown in Figure 6a. The piezoresistive elements are located in the stress concentration regions of the resonant beams. When the resonant beam vibrates, the piezoresistance undergoes periodic changes, thereby converting mechanical vibration into an electrical output signal. Excessive deviation in piezoresistance values across chips on the wafer would directly lead to degraded sensor consistency or even malfunction [26,27]. According to the experimental results, from the perspective of data distribution, the piezoresistance values of the five sample chips ranged from 143.4 Ω to 145.1 Ω, with an average value of 143.96 Ω, a standard deviation of 0.5919 Ω, and a relative deviation of merely 0.411%. This indicates that the wafer’s piezoresistor doping process exhibits high uniformity, with stable control over ion implantation dose and annealing process parameters, effectively mitigating the impact of resistance drift on sensor consistency.

(2) Drive Beam Comb-Drive Isolation Test (Test Path: P1–P3)

The purpose of the drive beam comb-drive isolation test is to verify the completeness of the etching process between drive electrodes. In this study, the isolation status between comb-drive electrodes was assessed by measuring the resistance value across test path P1–P3. In a resonant pressure sensor, the comb-drive electrodes (interdigitated structures) on the drive beams must remain electrically isolated from one another. Incomplete etching resulting in adhesion between adjacent comb fingers would cause a short circuit in the drive signal, preventing the generation of effective electrostatic force excitation on the resonant beam [28]. The test results indicate that the P1–P3 path for all samples exhibited an open-circuit state (resistance values exceeding the MΩ range), demonstrating that the etching between comb-drive electrodes is complete and free of adhesion, and that the drive structure can properly receive excitation signals. This validates that the wafer’s deep silicon etching process is capable of fabricating the comb-drive structures while maintaining electrical isolation.

The results of the aforementioned two wafer-level electrical tests confirm that the resonant pressure sensor wafer possesses excellent performance in terms of piezoresistance uniformity and electrical isolation of the drive structures, thereby establishing a foundation for subsequent frequency response testing.

### 3.2. Intrinsic Frequency Characterization of the MEMS Resonant Pressure Sensing Module

Following the verification of wafer-level electrical characteristics, frequency sweep testing was further conducted on the resonant pressure sensor using a Keysight E5061B network analyzer (Keysight Technologies Co., Ltd., Shanghai, China.) to determine its intrinsic resonant frequency range. The test was performed at room temperature (25 °C) with a sweep frequency ranging from 5 kHz to 50 kHz. The measured results are presented in Figure 6b.

As observed from the measurement results, the amplitude response across the entire swept frequency range can be divided into three distinct frequency bands. In the low-frequency band (5–15 kHz), the amplitude exhibits severe fluctuations with pronounced noise and spurious responses. These phenomena primarily originate from parasitic capacitances of the chip, lead inductances, and low-frequency parasitic effects associated with the test fixture, and do not reflect the intrinsic resonant characteristics of the sensor. Therefore, this band is excluded from subsequent analysis. In the mid-frequency band (20–35 kHz), a prominent amplitude peak appears in the measurement curve, corresponding to the intrinsic resonance peak of the resonant pressure sensor. Within this frequency range, the mechanical vibration mode of the sensor structure is effectively excited, achieving the highest energy coupling efficiency and representing the core operating frequency interval of the device. In the high-frequency band (35–45 kHz), the amplitude response gradually stabilizes, fluctuations diminish, and the signal approaches the noise floor. This indicates that once the excitation frequency exceeds the intrinsic resonant frequency, the energy coupling efficiency of the sensor declines significantly, and the sensitive structure no longer sustains an effective resonant state.

**Figure 7:**
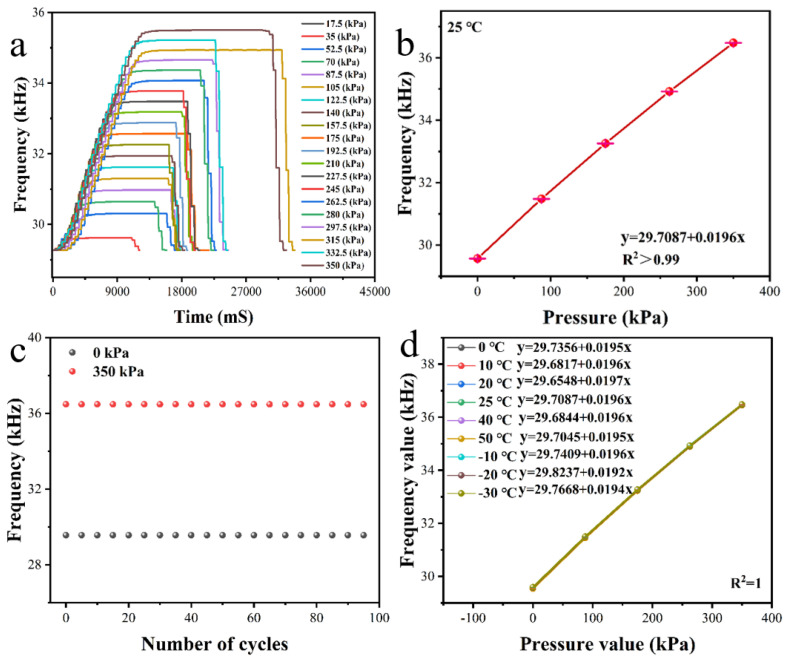
Pressure–frequency response characteristics of the MEMS resonant pressure sensor: (**a**) Sensor output frequency response curves over time under different applied pressures; (**b**) Relationship between sensor output frequency and applied pressure at room temperature, with linear fitting result; (**c**) Comparison of full-scale output and zero baseline after 1000 pressure cycles at 25 °C; (**d**) Pressure–frequency response characteristic curves of the sensor at different temperatures.

Based on the comprehensive test results and analysis, the intrinsic resonant frequency range of the resonant pressure sensor module under the present test conditions is approximately 20–35 kHz. This result provides a critical basis for the rational selection of the excitation signal frequency in subsequent pressure–frequency response characterization, thereby ensuring a stable and highly sensitive output response from the sensor within its operational frequency band. For reference, the resonant peak frequency under zero applied pressure is around 29.3 kHz (29.268 kHz); the shift of this peak with applied pressure is presented in Figure 7b.

### 3.3. Pressure–Frequency Response Characteristics of the MEMS Resonant Pressure Sensing Module

Following the wafer-level electrical characterization, a systematic investigation of the pressure–frequency response was conducted. A high-precision pressure controller was employed to apply a linear pressure ramp from 0 to 350 kPa to the sensor, while the resonant frequency output was synchronously recorded. The test was performed at room temperature (25 °C). The measured results are shown in Figure 7a. The frequency output of the MEMS resonant pressure sensing module initially increased linearly and subsequently reached a steady-state frequency plateau. The final output frequency varied according to the applied pressure level. It should be noted that the observed frequency response time in this measurement does not represent the intrinsic response time of the device to real-time barometric changes; rather, it is a consequence of the finite rate of pressure change imposed by the pneumatic tubing system during the test.

Aggregating the test results across different pressure levels, the sensor’s resonant frequency exhibits a well-defined monotonic increasing trend with applied pressure, and the overall curve remains smooth without noticeable inflection points or abrupt discontinuities. This indicates that the sensitive structure experienced neither plastic deformation nor resonant mode switching within the tested pressure range. Over the full-scale range of 0–350 kPa, the frequency response spans from 29.268 kHz to 35.4975 kHz, corresponding to a total frequency shift of 6.2295 kHz and an average sensitivity of approximately 0.02 kHz/kPa. As illustrated in Figure 7b, linear regression performed on the data points yields a pressure–frequency relationship of y = 0.0196x + 29.7087, with a coefficient of determination R^2^ exceeding 0.999, demonstrating excellent linearity of the sensor across the full measurement range [29].

However, to achieve the targeted ultra-high accuracy of 0.009% FS over the entire operating temperature range (−30 °C to 50 °C) and full pressure span (0–350 kPa), the residual nonlinearity and temperature-induced drift must be further compensated. Therefore, a dedicated temperature-dependent compensation algorithm is adopted, as detailed in Section 3.4.

After undergoing 1000 pressure cycles at 25 °C, the sensor maintained favorable repeatability. The full-scale output and zero baseline after 1000 cycles are presented in the table within Figure 7c (only a representative subset is plotted). Both the full-scale output and the zero baseline exhibited negligible drift, with a repeatability error of less than 0.008% FS. This result indicates that the sensitive structure did not suffer fatigue damage or performance degradation under cyclic pressure loading. Furthermore, we systematically characterized the mechanical hysteresis of the sensor by measuring the output frequency deviation during pressure ascend and descend cycles (0 → 350 → 0 kPa). The maximum mechanical hysteresis error was found to be less than 0.00050 kHz, demonstrating negligible backlash in the resonant structure (Table S2). The thermal hysteresis was also evaluated by subjecting the sensor to temperature cycles between −30 °C and 50 °C without pressure loading; the corresponding frequency output shift due to thermal hysteresis remained within 0.00375 kHz (Table S3). These hysteresis characterizations further confirm that the mechanical stiffness of the resonant system and its electrical characteristics remain highly consistent. This establishes a solid foundation for the reliable deployment of the sensor in long-term dynamic pressure monitoring applications [30].

Furthermore, to evaluate the pressure–frequency response characteristics of the MEMS resonant pressure sensing module under varying temperature environments, tests were conducted over a temperature range from −30 °C to 50 °C. The results are presented in Figure 7d. The comprehensive test data reveal that the pressure–frequency response curves of the sensor exhibit outstanding consistency and stability across this wide temperature span, with the output characteristics at different temperatures nearly overlapping. These demonstrate that the MEMS resonant pressure sensing module possesses robust temperature immunity, thereby providing a stable and reliable pressure–frequency response baseline for operation across diverse ambient thermal conditions [31,32]. However, to achieve the targeted ultra-high accuracy of <0.01% FS, further compensation of residual nonlinearities and temperature drift is required, as detailed in Section 3.4.

### 3.4. Temperature Compensation Algorithm and Pressure Calculation Implementation

Although the sensor exhibits excellent quasi-linearity at room temperature (R^2^ > 0.999, as shown in Section 3.3), achieving the target accuracy of <0.01% FS over the full temperature range (−30 °C to 50 °C) requires correction of residual nonlinearities and temperature drift. A third-order polynomial model with temperature-dependent coefficients is therefore implemented in the MCU. The compensated pressure P is calculated as Equation (1):

(1) $$P=A\left(T\right){\;f}^{3}+B\left(T\right){\;f}^{2}+C\left(T\right)\;f+D\left(T\right)$$

where *f* is the measured resonant frequency (kHz) after ADC sampling and filtering, and T is the real-time ambient temperature (°C). The coefficients *A*(*T*), *B*(*T*), *C*(*T*), and *D*(*T*) are themselves functions of temperature, determined through a two-step calibration process.

Step 1: Coefficient extraction at discrete temperature points.

Calibration is performed at N = 6 representative temperatures: −30 °C, −20 °C, 0 °C, 10 °C, 25 °C, and 50 °C. At each fixed temperature T_j_, the sensor is subjected to a series of known pressure levels (e.g., 0, 50, 100, 150, 200, 250, 300, 350 kPa), and the corresponding output frequencies are recorded. For each T_j_, a third-order polynomial *P = A_j_ f*^3^ + *B_j_ f*^2^ + *C_j_ f* + *D_j_* is fitted to the pressure-frequency data using the least-squares method, minimizing the residual sum of squares as Equation (2):

(2) $$min\sum_{i}{\left[P_{i}-(A_{j}f_{i}^{3}+{B_{j}f}_{i}^{2}+C_{j}f_{i}^{1}+D_{j})\right]}^{2}$$

This yields a discrete coefficient table {(T_j_, A_j_, B_j_, C_j_, D_j_)} stored in the MCU Flash.

Step 2: Continuous temperature modeling.

To obtain smooth coefficient variation with temperature, each coefficient is further fitted as a third-order polynomial of *T*, as Equations (3)–(6):

(3) $$A\left(T\right)=a_{3}{\;T}^{3}+a_{2}{\;T}^{2}+a_{1}\;T+a_{0}$$

(4) $$B\left(T\right)=a_{3}{\;T}^{3}+a_{2}{\;T}^{2}+a_{1}\;T+a_{0}$$

(5) $$C\left(T\right)=a_{3}{\;T}^{3}+a_{2}{\;T}^{2}+a_{1}\;T+a_{0}$$

(6) $$D\left(T\right)=a_{3}{\;T}^{3}+a_{2}{\;T}^{2}+a_{1}\;T+a_{0}$$

Using the six discrete points (*T_j_*, *A_j_*), (*T_j_*, *B_j_*), etc., the coefficients $a_{3}\ldots a_{0}$, $b_{3}\ldots b_{0}$, $c_{3}\ldots c_{0}$, $d_{3}\ldots d_{0}$ are determined via least-squares regression (solving a 4 × 4 normal equation system with Gaussian elimination). These 16 polynomial coefficients are pre-computed offline and permanently stored in Flash.

Online compensation: During real-time operation, the MCU reads the current temperature T, evaluates the four cubic polynomials to obtain A(T), B(T), C(T), and D(T), and then computes the final pressure P using the third-order model.

**Figure 8:**
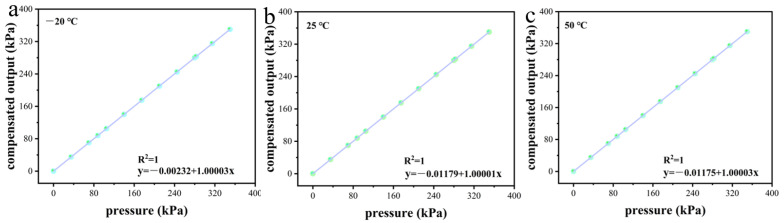
Typical output characteristics of the compensated MEMS resonant pressure sensor at three representative temperatures: (**a**) −20 °C, (**b**) 25 °C, and (**c**) 50 °C.

After implementing the temperature compensation algorithm described above, the sensor achieves a measurement accuracy of 0.009% FS (full-scale reference error, δ FS = ΔP/FS × 100%, where ΔP is the absolute error between the measured pressure and the reference) over the full pressure range (0–350 kPa) across the entire operating temperature span from −30 °C to 50 °C. This value represents the calibrated accuracy after compensation. Figure 8 illustrates typical output characteristics of the compensated sensor at three representative temperatures: −20 °C, 25 °C, and 50 °C. As shown, the pressure–frequency responses at these temperatures exhibit excellent linearity and consistency, with the compensated output closely matching the applied pressure values across the entire scale. The statistical analysis covers the full temperature and pressure ranges, both ascending and descending pressure cycles, and no data points were excluded as outliers. These results confirm that the adopted third-order polynomial compensation model effectively corrects residual nonlinearities and temperature-induced drift, thereby ensuring ultra-high accuracy under varying thermal environments.

### 3.5. Performance Comparison

To better evaluate the comprehensive performance of the sensor developed in this work, a comparison was made with current mainstream resonant pressure sensors and typical MEMS piezoresistive sensors. The comparison covers five aspects: sensing principle, measurement range, sensitivity/accuracy, repeatability, and operating temperature range. The results are presented in Table 1.

The comparison shows that the accuracy of the proposed sensor (0.009% FS) is comparable to that of high-end resonant sensors (±0.01% FS) and substantially superior to that of piezoresistive sensors (±0.5% FS). Moreover, the sensor exhibits excellent repeatability (≤0.008% FS). It is worth noting that, compared with conventional detection schemes, the piezoresistive detection approach adopted herein offers reduced circuit complexity, thereby facilitating system integration. These results validate the high performance and practical utility of the proposed approach.

### 3.6. Atmospheric Pressure Measurement Experiment

**Figure 9:**
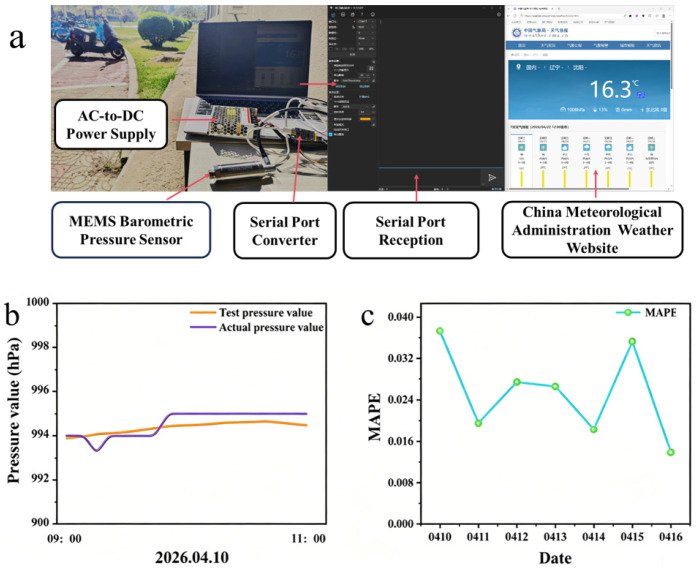
(**a**) Actual test scene for atmospheric pressure measurement, Liaoning University, 10–16 April 2026; (**b**) Time-series plot of a typical actual atmospheric pressure measurement result; (**c**) MAPE values for different test days.

Figure 9a shows the actual test setup during the experimental period. Figure 9b presents a time-series comparison between the measured atmospheric pressure values and the actual atmospheric pressure values recorded by the meteorological station on a typical day. As can be observed, the two curves exhibit highly consistent variation trends and closely matched numerical values, demonstrating the favorable dynamic tracking capability of the sensing system.

To quantitatively evaluate the measurement accuracy, the mean absolute percentage error (MAPE) was adopted as the evaluation metric, defined as Equation (7):

(7) $$MAPE=\frac{1}{n}\sum_{i=0}^{n}\frac{\left\|P_{Mi}-P_{Ri}\right\|}{P_{Ri}}$$

where I denotes the specific data point index, n is the total number of data points, and PMi and Pri represent the measured and reference atmospheric pressure values at the i-th data point, respectively.

Figure 9c summarizes the MAPE values for each test day. Throughout the entire test period, the MAPE values for all test days remained below 0.05%, maintaining a consistently low level. This indicates that the input–output characteristic of the sensing system exhibits good linearity and is capable of sustaining stable measurement accuracy. This result also indirectly validates the effectiveness of the compensation algorithm described in Section 3.4, as the system maintains high accuracy under real-time, varying ambient conditions.

It should be noted that the MAPE values obtained in this experiment differ numerically from the full-scale accuracy determined through laboratory calibration. This discrepancy primarily arises from the differing reference benchmarks employed. Such differences may be attributed to factors including the geographical separation between the test site and the meteorological station, as well as ambient temperature fluctuations.

To further assess whether the difference between the measured and reference values stems from systematic offset, a paired-sample *t*-test was performed on the two datasets. The result yields P = 0.8634, indicating no statistically significant difference between the two groups. In summary, the pressure sensing system exhibits high accuracy and reliability during the one-week measurement period in practical atmospheric pressure measurement, with no substantive discrepancy between the official reference data and the test results. Extended-duration field tests will be conducted in future work to evaluate long-term drift characteristics further.

## 4. Conclusions

Addressing the demand for high-precision pressure measurement, this paper has presented the design and implementation of a MEMS resonant pressure sensor based on electrostatic excitation and piezoresistive detection.

The experimental results demonstrate that over the operating temperature range from −30 °C to 50 °C, the developed sensor achieves favorable measurement accuracy (0.009% FS) and excellent repeatability (≤0.008% FS) across the pressure range of 0–350 kPa. Furthermore, real-time atmospheric pressure monitoring results show a mean absolute percentage error of less than 0.05% compared with the reference atmospheric pressure data for Shenyang, China, provided by the China Meteorological Administration, further demonstrating the sensor’s potential for engineering practicality in dynamic pressure measurement scenarios.

This work provides a system-level solution for high-precision, high-stability MEMS resonant pressure sensors, with promising application prospects in real-time barometric monitoring, aerospace, and industrial control.

## Figures and Tables

**Table 1:** Performance comparison of the sensor.

Sensing Principle	Range	Accuracy	Repeatability	Operating Temp. Range	Calibration Method	Before/After Compensation	Sensor Size	Literature
Electrostatic excitation + piezoresistive detection resonant type	0–350 kPa	0.009% FS	≤0.008% FS	−30 °C to 50 °C	polynomial	After compensation	4.8 × 4.8 × 3.8 mm	This work
Volume compression sensitivity + dual resonator	0.1–70 MPa	≤0.01% FS	≤0.01% FS	−10 °C to 50 °C	polynomial	After compensation	3.3 × 3.3 × 1.6 mm	[2]
Electrostatic excitation + piezoresistive detection resonant type	0–200 kPa	0.5% FS	≤0.01% FS	−40 °C to 80 °C	polynomial	After compensation	4.7 × 5.7 mm	[33]
Single resonator, amplitude-based temperature compensation	10–100 kPa	±0.012% FS	0.009% FS	−20 °C to 60 °C	polynomial	After compensation	Not specified	[9]
Electrostatic excitation + capacitive detection	20–280 kPa	±0.02% FS	0.01% FS	−40 °C to 80 °C	Not specified	Not specified	Not specified	[34]
Electromagnetic excitation + electromagnetic detection	100–1000 kPa	0.111% FS	0.01% FS	−45 °C to 65 °C	polynomial	After compensation	10 × 10 mm	[35]
Electrostatic stiffness modulation	10–200 kPa	±0.02% FS	0.01% FS	−55 °C to 125 °C	polynomial	After compensation	Not specified	[10]

## Data Availability

Data are contained within the article.

## Supplementary Materials

The following supporting information can be downloaded at https://www.mdpi.com/article/10.3390/mi17060717/s1. Figure S1: Signal processing and data conversion flowchart of the MEMS resonant pressure sensor system; Figure S2: PCB layout of the MEMS resonant pressure sensor; Figure S3: Circuit schematic of the MEMS resonant pressure sensor; Figure S4: Schematic diagram of the pickup circuit module; Figure S5: Schematic diagram of the excitation circuit module; Table S1: Key specifications of the developed MEMS Resonant Pressure Sensing Module; Table S2: Mechanical hysteresis; Table S3: Thermal hysteresis.
