# Supplementary material for: High-Precision MEMS Resonant Pressure Sensor for Real-Time Barometric Monitoring
Source: Micromachines (Basel). 2026 Jun 12;17(6):717. doi: 10.3390/mi17060717 (PMC13302947; doi:10.3390/mi17060717)
Supplement: Supplementary file 1 [file micromachines-17-00717-s001.zip › micromachines-4359311-supplementary.pdf]

High-Precision MEMS Resonant Pressure Sensor for Real-Time Barometric Monitoring

Fei Xia[1,†], Shuang Pang[1,†], Yutong Bai[1], Zishuai Zhang[1], Lulu Feng[1], Yizheng Hou[1], Yuxiang Wang[1], Zhiyu Liu[1], Yifei Sun[2], Jiwei Wang[1], Shiyu Wang[1,*]

a

1 College of Physics, Liaoning University, Shenyang, 110036, China
2 College of Information Engineering, Shenyang University of Chemical Technology, Shenyang 110142, China
* E-mail: wangshiyu@lnu.edu.cn (Shiyu Wang)
† These authors contributed equally to this work.

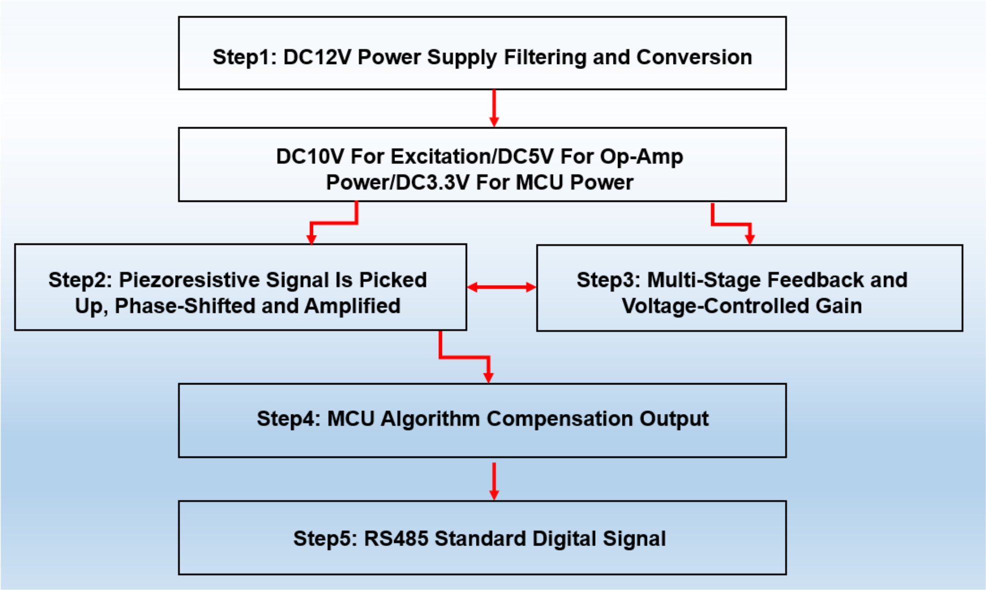

Figure S1. Signal processing and data conversion flowchart of the MEMS resonant pressure sensor system.

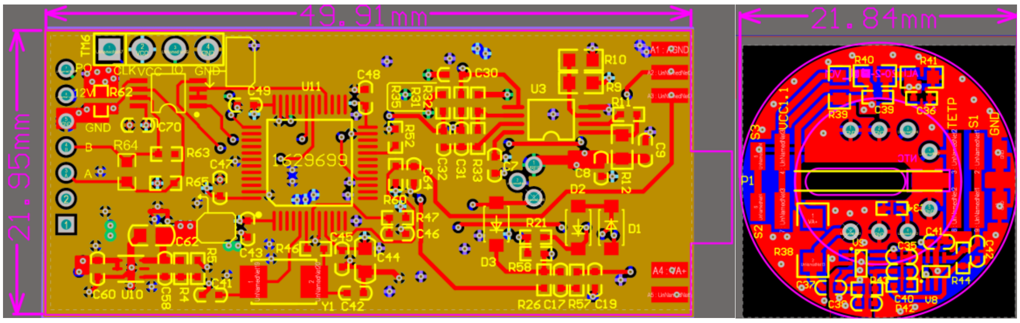

Figure S2. PCB layout of the MEMS resonant pressure sensor.

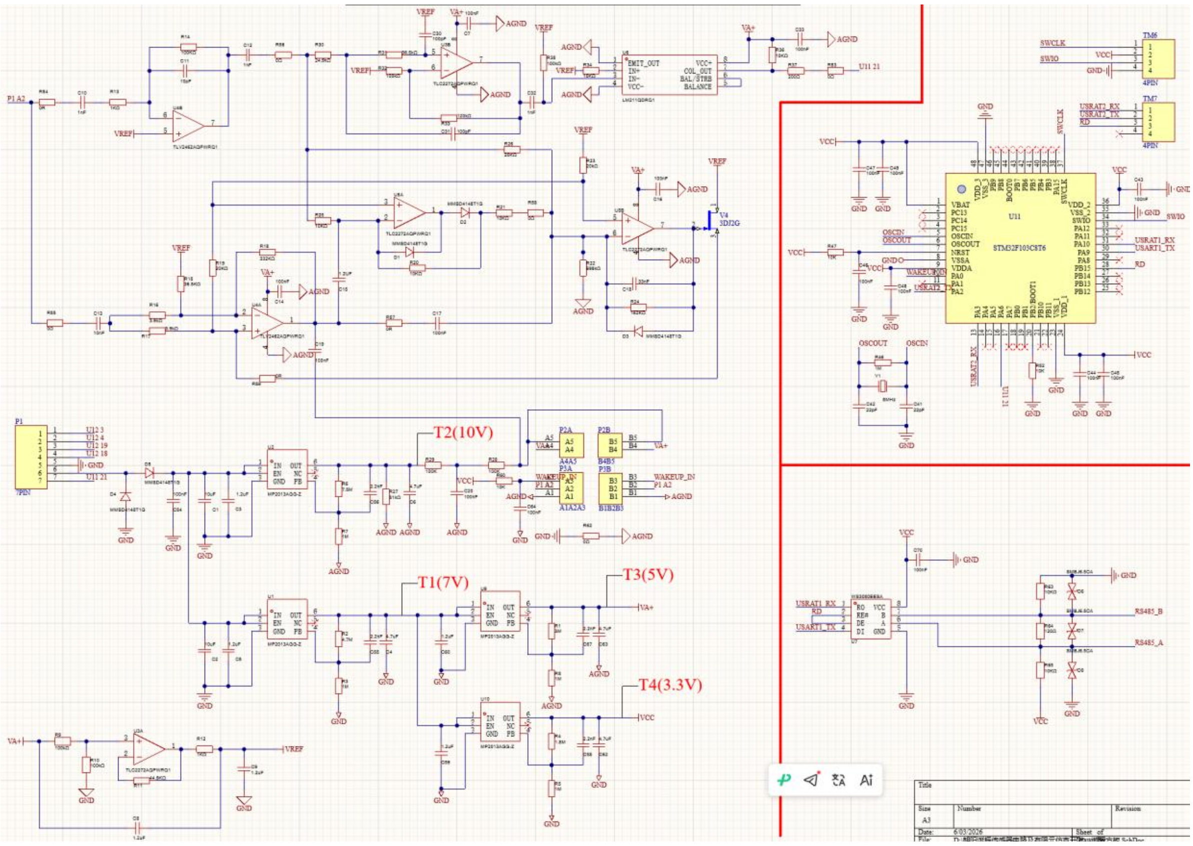

Figure S3. Circuit schematic of the MEMS resonant pressure sensor.

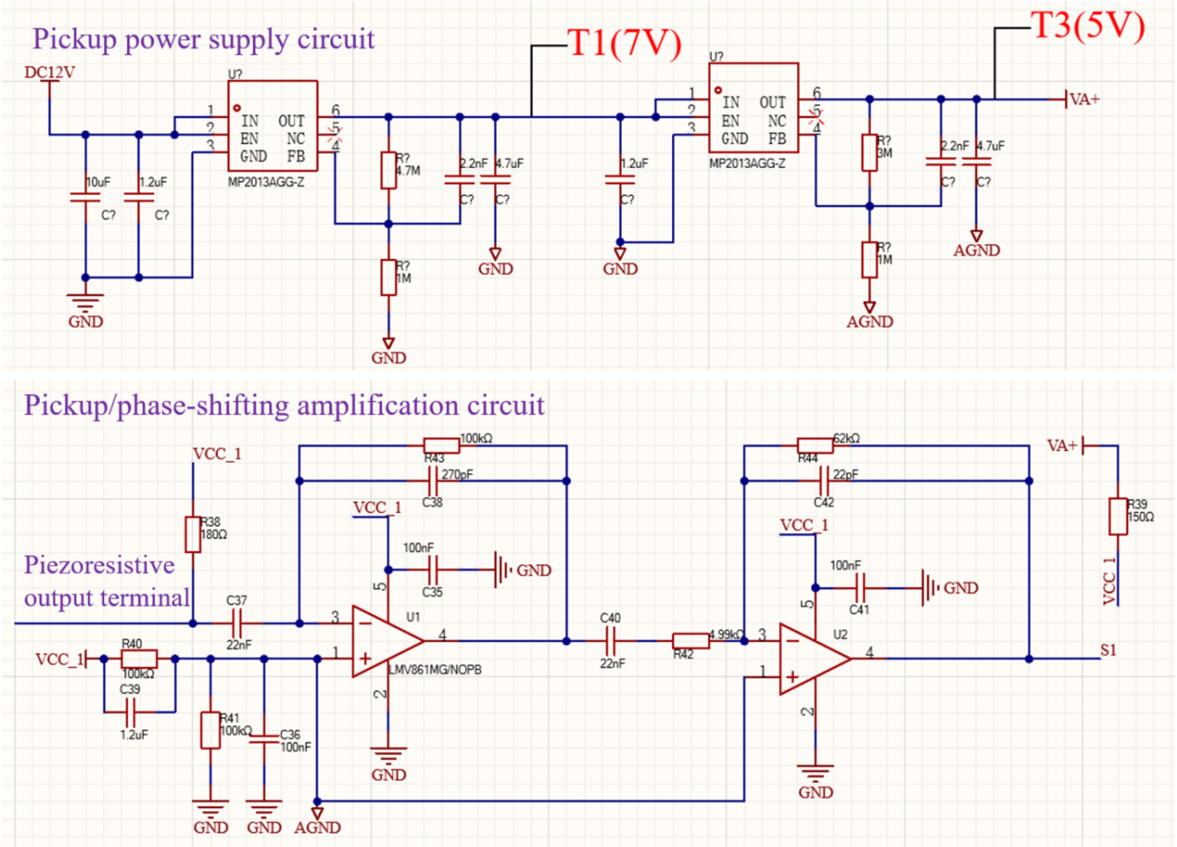

Figure S4. Schematic diagram of the pickup circuit module.

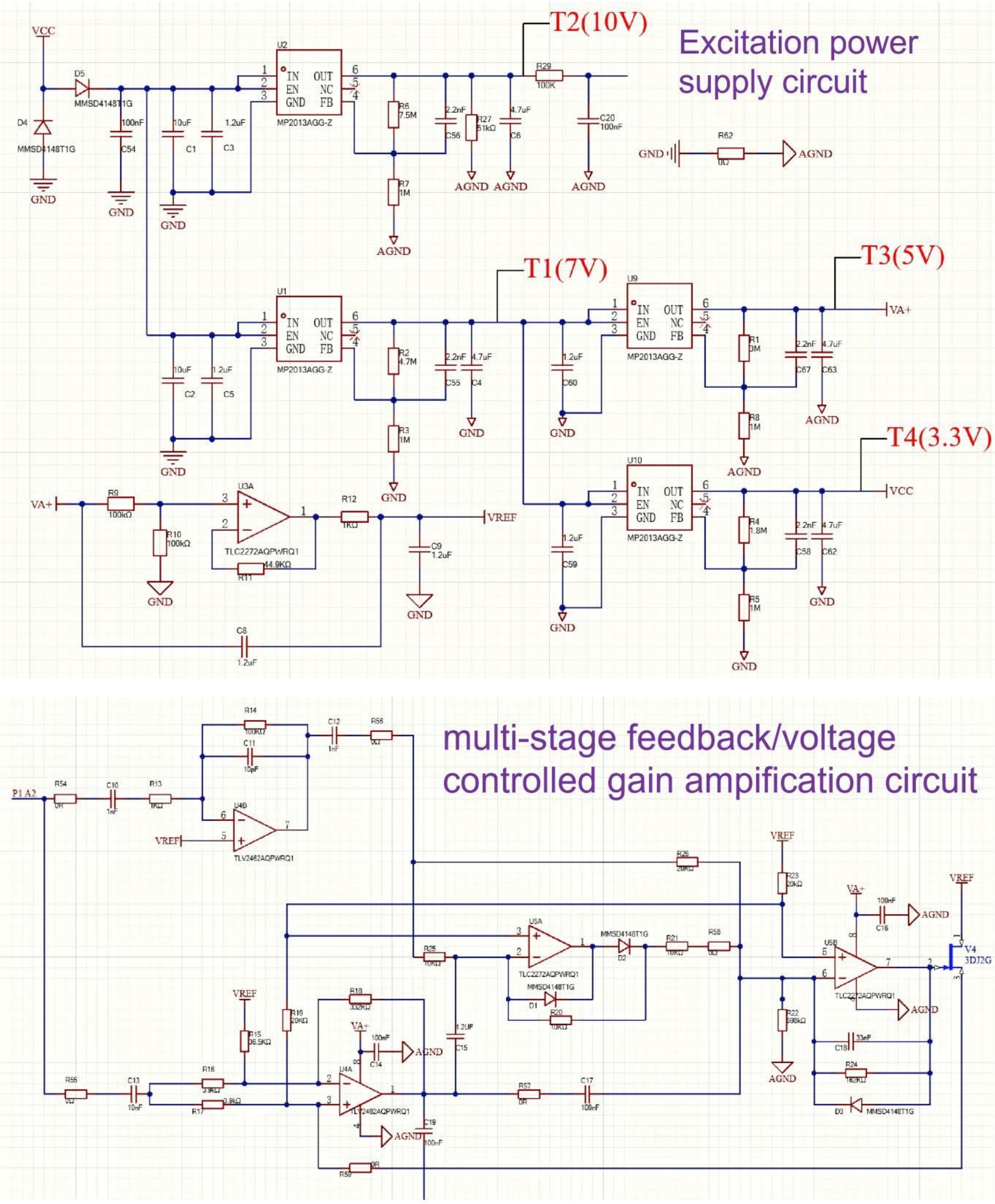

Figure S5. Schematic diagram of the excitation circuit module.

Table S1. Key specifications of the developed MEMS Resonant Pressure Sensing Module.

Parameter	Value / Description
Pressure range	0 – 350 kPa
Proof pressure	525 kPa
Burst pressure	875 kPa
Supply voltage	12 VDC
Supply current	< 5 mA
Output frequency at 0 kPa (over -30 °Cto 50 °C)	29.7668 kHz to 29.7045 kHz
Output frequency at 350 kPa (over -30 °C to 50 °C)	36.4835 kHz to 36.4536 kHz
Insulation resistance (body to ground)	>100 MΩ @ 100 VDC
Operating temperature	-30 °C to 50 °C
Outer diameter	15.8 mm
Length	8.5 mm (core body); 62 mm (with PCB)
Weight	~26 g

Parameter	Value / Description
Housing material	316L stainless steel
Pressure inlet	Female G1/4 internal thread
Electrical pinout	S1: vibration pickup; S2: excitation; Temp: temperature measurement; A: RS485A; B: RS485B;12 V: power; GND: body ground;

Table S2. Mechanical hysteresis.

Stroke	Pressure Point (kPa)							
	0	50	100	150	200	250	300	350
Forward Stroke	29.2875	30.3025	31.2785	32.2190	33.12625	34.00375	34.85175	35.6735
Reverse Stroke	29.2875	30.3020	31.27825	32.21875	33.12625	34.0035	34.8520	35.6735
Difference	0.0000	0.0005	0.00025	0.00025	0.0000	0.00025	-0.00025	0.0000

Table S3. Thermal hysteresis.

Temperature (℃)	Pressure (kPa)	Temperature Rising Cycle (-30 ~ 50 ℃)	Temperature Falling Cycle (50 ~ -30 ℃)	Cycle Difference (Rising - Falling)
-30	0	29.30750	29.30650	0.00100
-20	0	29.30475	29.30450	0.00025
-10	0	29.30075	29.30075	0.00000
0	0	29.29700	29.29675	0.00025
10	0	29.29650	29.29275	0.00375
20	0	29.29125	29.28800	0.00325
30	0	29.28625	29.28375	0.00250
40	0	29.28050	29.27925	0.00125
50	0	29.27425	29.27425	0.00000
